# Anticoagulation Reversal in Intracerebral Hemorrhage: A Case Report on the Efficacy of Andexanet Alfa in an Apixaban-Treated Patient

**DOI:** 10.7759/cureus.74750

**Published:** 2024-11-29

**Authors:** Corina Roman, Romeo Mihaila, Cosmin Nicodim Cindea, Antonia Iliescu, Iulian Roman-Filip, Anca Rafila Stingaciu, Alexandru Breazu, Vicentiu Saceleanu

**Affiliations:** 1 Neurology, County Clinical Emergency Hospital of Sibiu, Sibiu, ROU; 2 Hematology, Lucian Blaga University of Sibiu, Sibiu, ROU; 3 Hematology, County Clinical Emergency Hospital of Sibiu, Sibiu, ROU; 4 Neurosurgery, County Clinical Emergency Hospital of Sibiu, Sibiu, ROU; 5 Neurosurgery, Lucian Blaga University of Sibiu, Sibiu, ROU; 6 Neurology, Emergency County Hospital Targu-Mures, Targu Mures, ROU

**Keywords:** andexanet alfa, anticoagulation reversal, apixaban, hematoma evacuation, ich guidelines, intracerebral hematoma, intracranial hemorrhage (ich)

## Abstract

Intracerebral hemorrhage (ICH) presents complex clinical challenges, particularly in patients receiving anticoagulation therapy. This case report discusses the management of acute ICH in a 60-year-old male patient on long-term apixaban therapy, who arrived at the emergency department with altered consciousness, right-sided hemiplegia, and mixed aphasia. Computed tomography (CT) imaging revealed a 70 ml left lenticular-capsular hematoma with significant mass effect, necessitating rapid intervention. Due to the patient’s deteriorating neurological condition, andexanet alfa was administered as a reversal agent for apixaban, effectively reducing anti-factor Xa (FXa) activity and enabling an urgent left temporal craniotomy for hematoma evacuation. Postoperatively, the patient received ICU monitoring, blood pressure management, and supportive care, showing gradual improvement in consciousness and sensory aphasia over several days. Despite initial right hemiplegia and motor aphasia requiring rehabilitation, the patient showed excellent progress at one month, now walking independently and managing daily self-care. This report highlights the potential of andexanet alfa for effective anticoagulation reversal in critical ICH cases while also addressing associated thrombotic risks. It underscores the need for a multidisciplinary approach and emphasizes the importance of further research to refine treatment strategies for anticoagulated patients with ICH.

## Introduction

Intracerebral hemorrhage (ICH) accounts for about 15% of strokes, with a high mortality rate of up to 50% in the first year [1]. Patients on oral anticoagulants (OAC) are especially at risk, facing greater hematoma expansion and mortality. Over two-thirds of survivors endure significant functional dependency, highlighting ICH's severe impact on quality of life. While the ICH score is useful for assessing 30-day mortality, it remains insufficiently validated for OAC-associated ICH, which poses unique prognostic challenges [2].

Anticoagulation therapy predicts hematoma expansion and mortality in ICH, with risk levels varying by anticoagulant type. Patients with non-vitamin K antagonist oral anticoagulant-related ICH (NOAC-ICH) often have smaller hematomas and less severe strokes than those with vitamin K antagonist-related ICH (VKA-ICH). In life-threatening bleeding, andexanet alfa can rapidly reverse factor Xa (FXa) inhibitor effects to allow urgent intervention [3].

Andexanet alfa use carries risks, as thrombotic events can occur, especially in patients already predisposed to thromboembolism, which is often the reason they are prescribed FXa inhibitors in the first place. Reversing FXa inhibition increases thrombotic risk, heightened by andexanet alfa’s inhibition of tissue factor pathway inhibitor (TFPI). The duration of this procoagulant effect in bleeding patients remains unclear, and laboratory measures like anti-factor FXa activity and endogenous thrombin potential (ETP) may not reliably predict thrombotic risk, underscoring the need for careful monitoring and timely anticoagulation resumption [4].

In acute ICH patients on FXa inhibitors, andexanet alfa has the potential to reduce hematoma expansion compared to usual care [5]. However, 30% experienced thromboembolic events (ischemic stroke or myocardial infarction) within four days post reversal. Large randomized trials are needed to assess whether preventing hematoma expansion improves outcomes, despite high thromboembolism rates.

This case report discusses a 60-year-old male patient who developed acute ICH while on apixaban, with andexanet alfa used as an emergency reversal agent. The report details the patient’s condition, the role of andexanet alfa in reversing anticoagulation, and the risks and limitations of its use. It emphasizes the need for personalized strategies in managing OAC-ICH and calls for further research to optimize treatment in this high-risk group.

## Case presentation

Clinical background and presentation

A 60-year-old male patient with a medical history significant for atrial fibrillation was on long-term anticoagulation therapy with apixaban (5 mg twice daily). He presented to the emergency department after a sudden onset of headache, confusion, slurred speech, and right-sided weakness, as reported by his spouse. Upon examination, he demonstrated right-sided hemiplegia (0/5 on the Medical Research Council (MRC) scale), expressive aphasia, and a decreased level of consciousness with a Glasgow Coma Scale (GCS) score of seven (E1 + M4 + V2), signaling a substantial neurological deficit. 

Diagnostic imaging and laboratory findings

**Figure 1:**
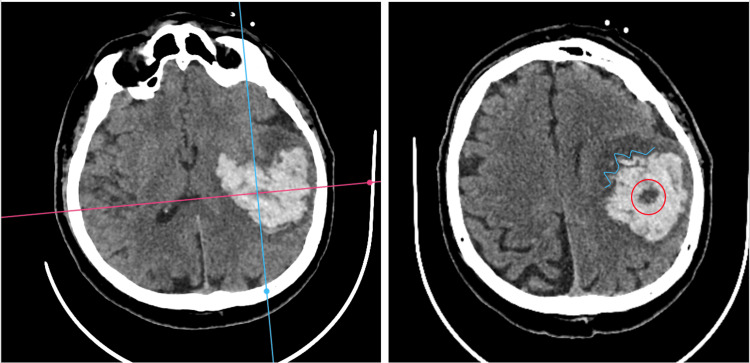
Cranial CT scan obtained at the emergency department Left: The head CT scan shows a 70 ml large left capuso-lenticular hematoma; Right: The head CT scan shows a black hole sign (red circle) and margin irregularities (light-blue line). Signs of active bleeding are noted.

Non-contrast computed tomography (CT) of the head (Figure 1) showed a large, acute left-sided lenticular-capsular intraparenchymal hemorrhage, approximately 70 ml in volume. The hematoma was associated with significant perilesional edema, midline shift of 5 mm, and effacement of the left lateral ventricle, indicating raised intracranial pressure (ICP). 

Given the life-threatening nature of the ICH and the black hole sign (swirl sign) on the CT scan, andexanet alfa was chosen for prompt reversal of anticoagulation, followed by a neurosurgical intervention.

Treatment approach: andexanet alfa administration and surgical intervention

The patient received andexanet alfa intravenously using the high-dose protocol recommended for rapid reversal in cases of life-threatening bleeding. An initial bolus was administered over 30 minutes at a target infusion rate of approximately 30 mg/min, followed by a continuous infusion at a rate of 8 mg/min for 120 minutes to maintain a therapeutic effect. The high-dose protocol was selected because the patient had taken the last dose of apixaban, 5 mg, just two hours before the onset of ICH.

**Figure 2:**
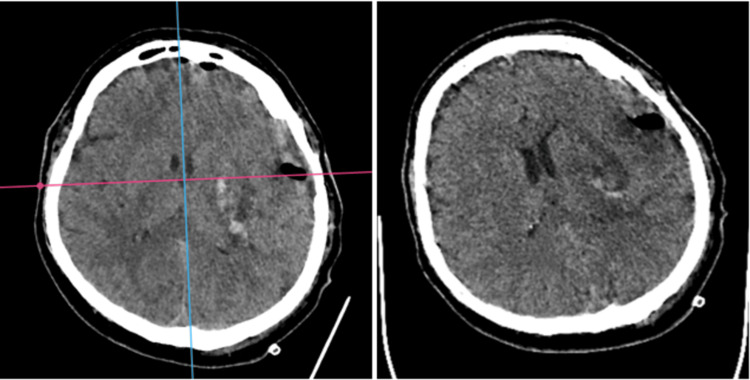
Postoperative cranio-cerebral CT obtained during follow-up evaluations A 24-hour postoperative CT scan (axial view) shows minimal residual hematoma with no recurrence of hemorrhage; no midline shift or mass effect is noted.

Following the administration of andexanet alfa, a left temporal craniotomy was performed to evacuate the hematoma and relieve ICP. Intraoperative findings revealed no active bleeding within the hematoma. Postoperative imaging (Figure 2) confirmed effective hematoma removal and a reduction in midline shift, which helped stabilize ICP.

Intensive care unit management and evolution

After surgery, the patient was transferred to the ICU for rigorous monitoring of neurological and cardiovascular status. Prophylactic measures to prevent thrombotic complications were implemented, including therapeutic doses of enoxaparin. In line with a precautionary clinical approach, a dose of 60 mg twice per day was selected, considering the patient's elevated risk factors for deep vein thrombosis (DVT) and the increased risk of thrombosis following Andexanet alfa administration. 

By the third postoperative day, the patient showed gradual neurological improvement. His GCS score increased to 12, and he was able to follow simple commands. Although aphasia and hemiplegia persisted, there was a slight improvement with early rehabilitative support, including passive and active-assisted physical therapy and speech therapy.

The total hospitalization costs for this patient amounted to 11,500 lei (approximately 2,300 euros) in the neurosurgery department for eight days, 15,000 lei (approximately 3,000 euros) in the neurology department for 16 days, and 14,000 lei (approximately 2,800 euros) in the rehabilitation department for 14 days, 8,100 euros in total. Emergency department costs, where andexanet alfa was administered, were not included. 

Outcome and follow-up

**Figure 3:**
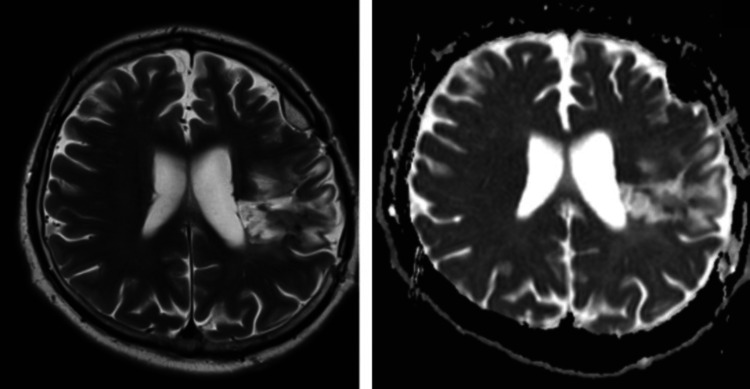
Brain MRI at the one-month follow-up T2-weighted imaging (left) and diffusion-weighted imaging (right) show a sequelae ischemic area located in the motor area of the frontal lobe, anterior to the central sulcus.

Over the next weeks, the patient showed improvement in motor skills but continued to experience significant dysarthria and hemiparesis. At the time of discharge to a rehabilitation facility for ongoing physical and speech therapy, muscle strength in the lower limb was 2/5 on the MRC Scale. After four weeks of rehabilitation, the patient demonstrated remarkable progress, regaining the ability to walk independently. Follow-up imaging (Figure 3) and potential reevaluation for resuming oral anticoagulation to prevent recurrent ischemic events were planned.

As a bilingual individual, the patient’s non-native language was less affected than the dominant one. He now tends to blend words from both languages, unintentionally switching between them, a code-switching phenomenon that has become more apparent during his recovery.

This favorable outcome highlights the potential for andexanet alfa in improving recovery in FXa inhibitor-associated ICH cases, although close monitoring for delayed thromboembolic events remains crucial.

Methods

Imaging was processed using RadiAnt DICOM Viewer (Medixant, Poznan, Poland) to analyze the hematoma. Volume (V) was calculated using the formula 𝑉=(𝑎×𝑏×𝑐)/2 where 𝑎, 𝑏, and c represent the hematoma's dimensions. Andexanet-alfa was administered for anticoagulation reversal, with an initial bolus and continuous infusion adjusted based on weight and FXa inhibitor dosage, monitored to prevent thromboembolic events. A craniotomy for hematoma evacuation was performed under general anesthesia, using a microscope for minimal invasivity, reducing mass effect and intracranial pressure, with imaging confirming complete evacuation. Rehabilitation included structured speech therapy for aphasia and physical therapy with active-assisted and passive exercises to improve motor function, strength, and mobility.

## Discussion

The presented case exemplifies the complex challenges of managing ICH in patients on FXa inhibitors. While anticoagulation therapy is essential for reducing thromboembolic risk in patients with conditions such as atrial fibrillation and previous ischemic stroke, it significantly heightens the likelihood of hemorrhagic events, particularly spontaneous ICH. This scenario underscores the delicate balance between the benefits of anticoagulation and the risks of bleeding, especially in older patients with multiple comorbidities.

The choice of andexanet alfa was driven by the urgent need for rapid reversal of apixaban, a common FXa inhibitor. Unlike traditional reversal agents such as prothrombin complex concentrate (PCC), andexanet alfa is specifically designed for FXa inhibitors, binding and inactivating them effectively to restore coagulation function within minutes [6]. Studies, including the Andexanet Alfa, a Novel Antidote to the Anticoagulation Effects of Factor Xa Inhibitors (ANNEXA-4) trial, demonstrate its efficacy in achieving hemostasis in severe bleeds, reinforcing its use in high-risk clinical scenarios like ICH. The availability of an antidote like andexanet alfa plays a pivotal role in the management of patients on anticoagulant therapy, enabling the reversal of anticoagulation effects and reducing the risk of uncontrolled bleeding. This capability is particularly critical in cases of ICH, where timely surgical intervention is essential to prevent worsening neuronal damage and increasing intracranial pressure. Without an antidote, the decision to proceed with prompt surgical intervention is usually approached with greater caution due to the high risk of intraoperative bleeding, potentially delaying life-saving procedures. The marked reduction in anti-factor Xa levels within minutes of administration aligns with evidence suggesting rapid and sustained reversal, providing the patient with the best chance for surgical recovery [7].

The decision to perform a left temporal craniotomy for hematoma evacuation highlights a vital component of ICH management: relieving ICP to prevent further brain injury. In this case, the pharmacological reversal facilitated by andexanet alfa achieved an acceptable coagulation status for surgery, thereby minimizing perioperative bleeding risk [8]. The patient’s postoperative recovery in the ICU underscores the importance of a multidisciplinary approach involving neurosurgeons, critical care specialists, and rehabilitation teams.

Despite substantial initial neurological impairment, the patient showed encouraging signs of recovery, indicating that timely intervention can mitigate the severe long-term effects of ICH [9]. Early improvements in GCS scores and motor function may reflect the combined effects of rapid anticoagulation reversal, surgical hematoma evacuation, and meticulous ICU management. However, ongoing impairments in aphasia and hemiplegia highlight that even with optimal management, ICH patients often require prolonged rehabilitative support to regain functionality and quality of life. This case highlights the potential role of andexanet alfa in the acute management of FXa inhibitor-associated ICH while emphasizing that comprehensive care, including ICU support, physical therapy, and speech therapy, is crucial for facilitating recovery [10].

Although andexanet alfa is a powerful tool, it carries a well-documented risk of thromboembolic events post-administration, as highlighted in several studies. Thrombotic events such as DVT and ischemic stroke have been reported in up to 10%-20% of patients treated with andexanet alfa, particularly within the first few days post infusion. The pathophysiological basis lies in andexanet’s strong affinity for FXa inhibitors, which can overcorrect the anticoagulant state and shift the hemostatic balance towards thrombosis. This risk necessitates cautious patient selection and close monitoring for signs of thrombosis, especially in high-risk patients with atrial fibrillation or a history of thrombotic events [11].

Cost considerations remain a significant limiting factor for the widespread use of andexanet alfa, as it is substantially more expensive than alternatives like PCC. Economic evaluations suggest that while andexanet alfa may be beneficial in high-risk bleeding situations, its routine use may not be financially sustainable, particularly in resource-limited settings. Therefore, its application should be reserved for cases where other interventions may not provide the rapid hemostasis required, as demonstrated in this case [12].

Continued research, including studies like the ANNEXA-I trial, is essential to evaluate the long-term safety and efficacy of andexanet alfa specifically in ICH patients. Further studies could refine treatment protocols, identify patients most likely to benefit and explore combined approaches with other agents. Developing reversal strategies that are both effective and cost-efficient remains a critical goal, as does assessing long-term outcomes in patients undergoing anticoagulation reversal [13].

We present a 60-year-old patient on chronic apixaban treatment who suffered a hemorrhagic stroke. The management of such cases entails clinical, economic, and social considerations. While the cost of Andexanet alfa is significant, it must be viewed in the context of long-term caregiving expenses and the broader implications for the patient’s quality of life and societal support systems. This case highlights the importance of balancing immediate medical needs with the financial and social impacts, emphasizing the need for a comprehensive and ethical approach to decision-making.

This case underscores the potential role of andexanet alfa in managing FXa inhibitor-associated ICH, highlighting its rapid reversal capabilities and facilitation of timely surgical intervention. Notably, the ANNEXA-4 trial demonstrated that andexanet alfa achieved effective hemostasis in 82% of patients with major bleeding events [14]. Additionally, a comparative study reported that andexanet alfa was associated with better hemostatic effectiveness and improved survival compared to four-factor prothrombin complex concentrate (4F-PCC) in patients with apixaban- or rivaroxaban-associated ICH [15]. However, this case report differs by providing a detailed account of individual patient management, emphasizing the importance of personalized treatment strategies and the need for further research to establish standardized protocols.

The administration of andexanet alfa, costing approximately 15,000 euros in Romania, necessitates a detailed cost-benefit analysis due to its significant upfront cost. The potential reduction in long-term caregiving expenses, which can amount to 500 euros monthly, justifies its use in patients likely to regain independence post-rehabilitation. Consequently, establishing specific inclusion and exclusion criteria is essential to optimize both clinical outcomes and economic efficiency, particularly given the variable prognosis in anticoagulation-related intracerebral hemorrhage. This strategy emphasizes the need for a methodical approach that integrates clinical and economic considerations [16].

The inclusion of andexanet alfa, costing approximately 15,000 euros per dose, significantly escalates the overall treatment costs, which already total 8,100 euros without it. This underscores the importance of its selective use, targeting patients most likely to derive substantial benefits from this high-cost intervention.

In developing countries like Romania, accessing andexanet alfa is challenging due to its sporadic availability, significantly straining healthcare systems. This limited access often renders the antidote an exception in clinical practice, highlighting the need for strategic healthcare planning to improve its distribution and ensure that it reaches patients in critical need.

## Conclusions

This case report illustrates the role of andexanet alfa as an effective reversal agent for managing ICH in anticoagulated patients on FXa inhibitors, such as apixaban. Timely administration led to rapid hemostasis and positive clinical outcomes, emphasizing the need for rapid intervention in life-threatening bleeds.

However, the increased thromboembolic risk post reversal necessitates a careful, patient-specific approach to managing anticoagulation resumption. The findings advocate for further research on andexanet alfa’s efficacy and safety in randomized controlled trials, which will help refine protocols for anticoagulated ICH patients, ensuring balanced decision-making that prioritizes both hemostasis and thrombotic risk management.
